# Supplementary material for: Demonstration of chemistry at a point through restructuring and catalytic activation at anchored nanoparticles
Source: Nat Commun. 2017 Nov 30;8:1855. doi: 10.1038/s41467-017-01880-y (PMC5707356; doi:10.1038/s41467-017-01880-y)
Supplement: Supplementary file 1 — Supplementary Information [file 41467_2017_1880_MOESM1_ESM.docx]

| **a** |
| --- |
| 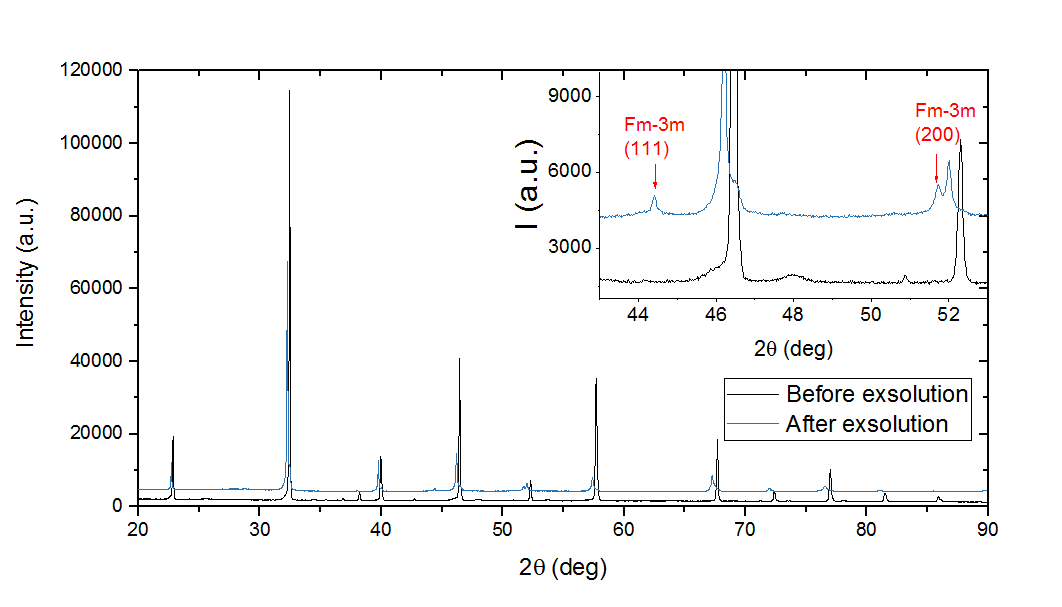 |
| **b** |
| 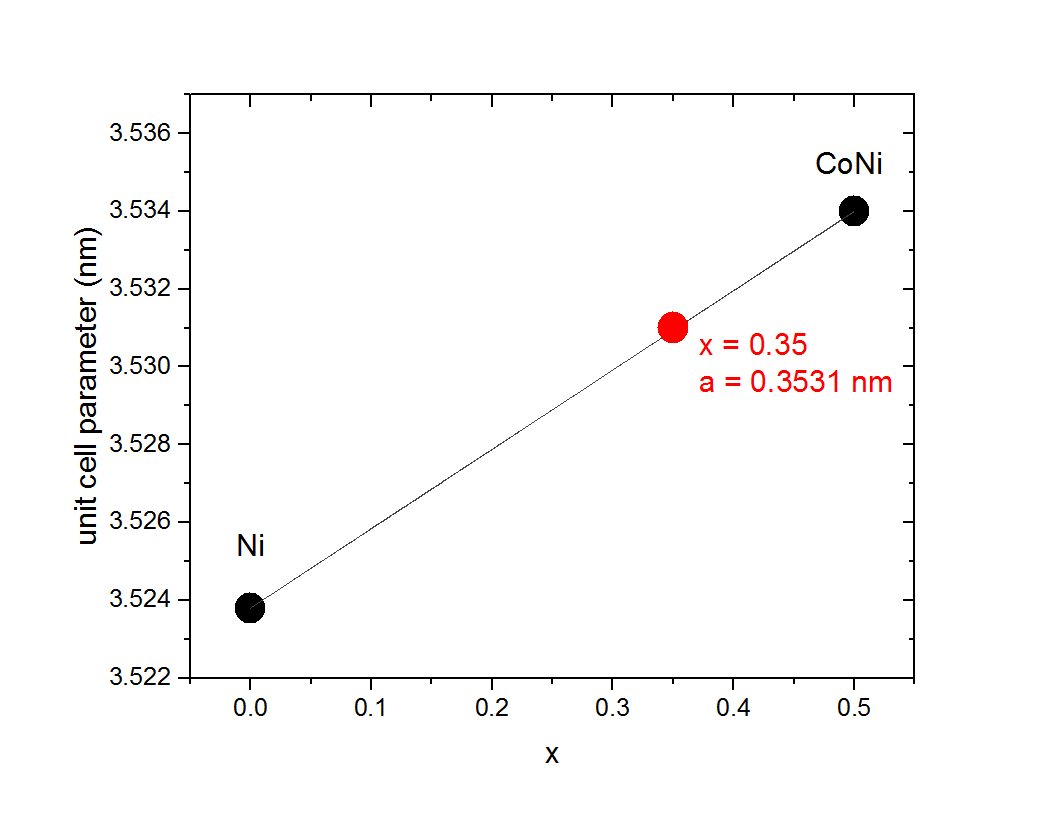 |

Supplementary Figure 1. Phase analysis for La_0.7_Ce_0.1_Co_0.3_Ni_0.1_Ti_0.6_O_3_.

(**a**) XRD patterns of the catalyst before and after exsolution of metal particles. The detail highlights the region in which the metal peaks (red) are visible. (**b**) Unit cell parameter as a function of composition for Co_1-x_Ni_x_ alloys also indicating the unit cell parameter and corresponding composition of the alloy obtained in this study as derived from the XRD pattern in (a).


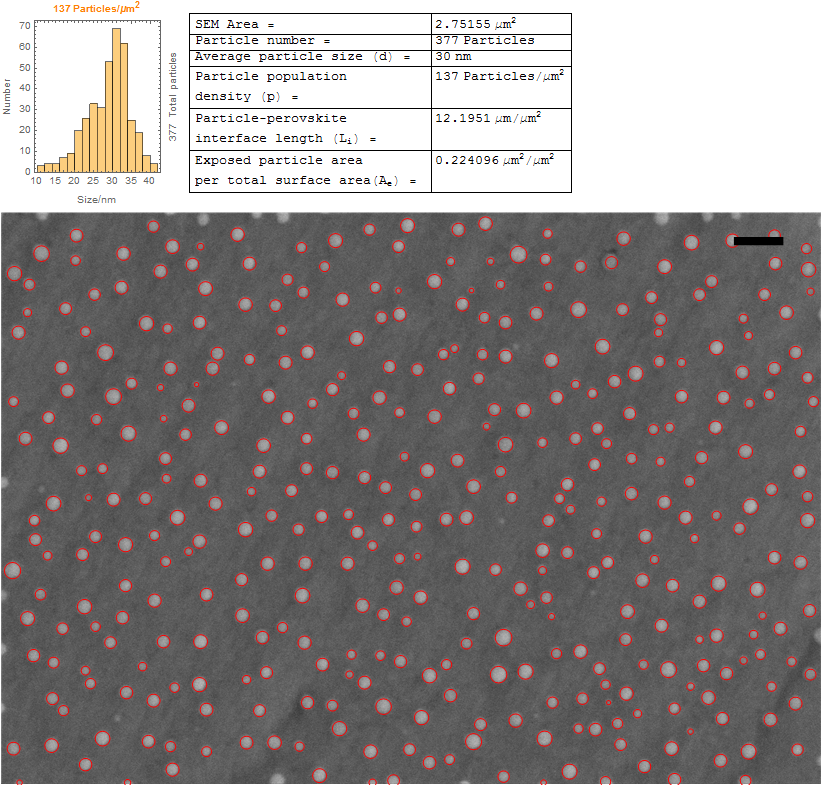


Supplementary Figure 2. Microstructure and particle size analysis for $\boldsymbol{Ni}{}_{\boldsymbol{P}}^{\boldsymbol{30}}$.

The particles are metallic and their average diameter is 30 nm. The corresponding metal particle loading is ~1.5∙10^‑2^ g of metal particles per m^2^ of catalyst surface area. Scale bar, 100 nm.


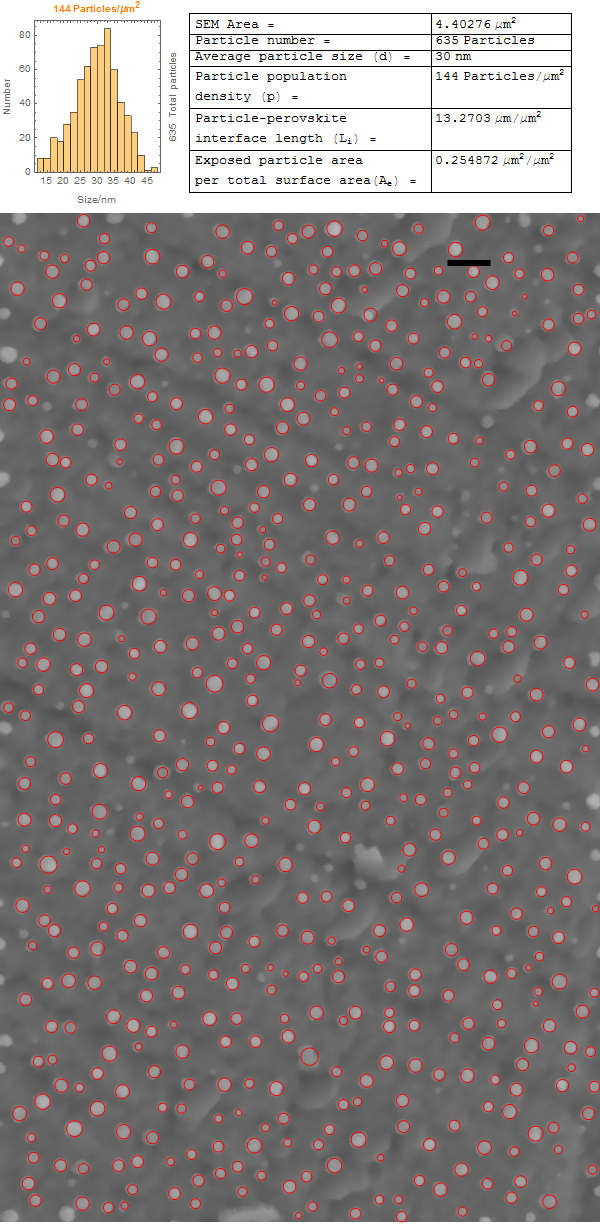


Supplementary Figure 3. Microstructure and particle size analysis for $\boldsymbol{CoNi}{}_{\boldsymbol{P}}^{\boldsymbol{30}}$.

The particles are metallic and their average diameter is 30 nm. The corresponding metal particle loading is ~2.1∙10^‑2^ g of metal particles per m^2^ of catalyst surface area. Scale bar, 100 nm.


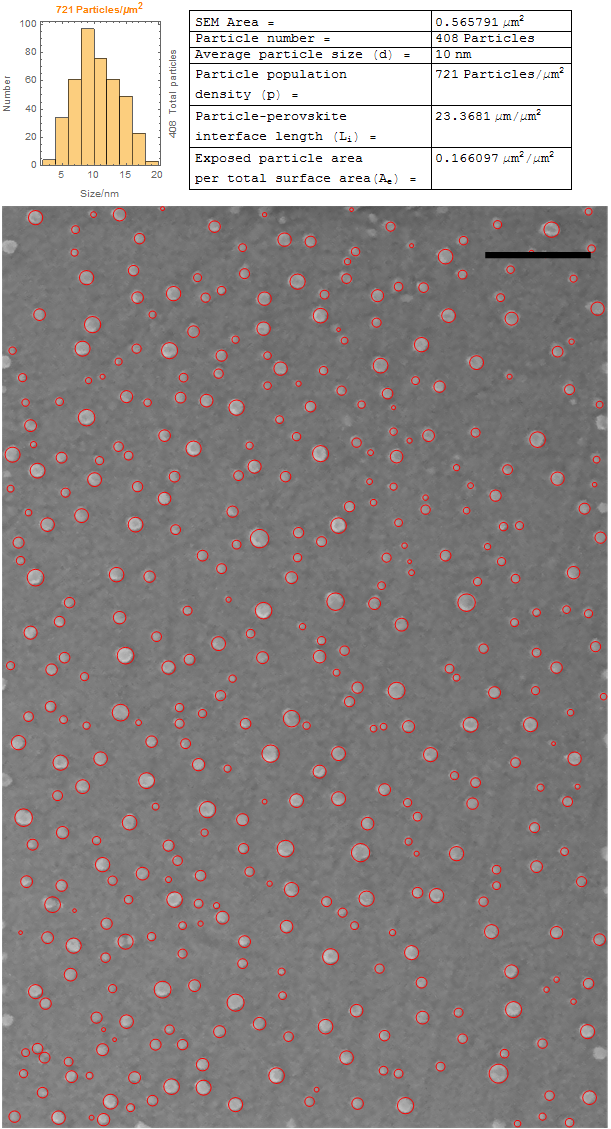


Supplementary Figure 4. Microstructure and particle size analysis for $\boldsymbol{CoNi}{}_{\boldsymbol{P}}^{\boldsymbol{10}}$.

The particles are metallic and their average diameter is 10 nm. The corresponding metal particle loading is ~5.1∙10^‑3^ g of metal particles per m^2^ of catalyst surface area. Scale bar, 100 nm.

| **a** | |  |
| --- | --- | --- |
| 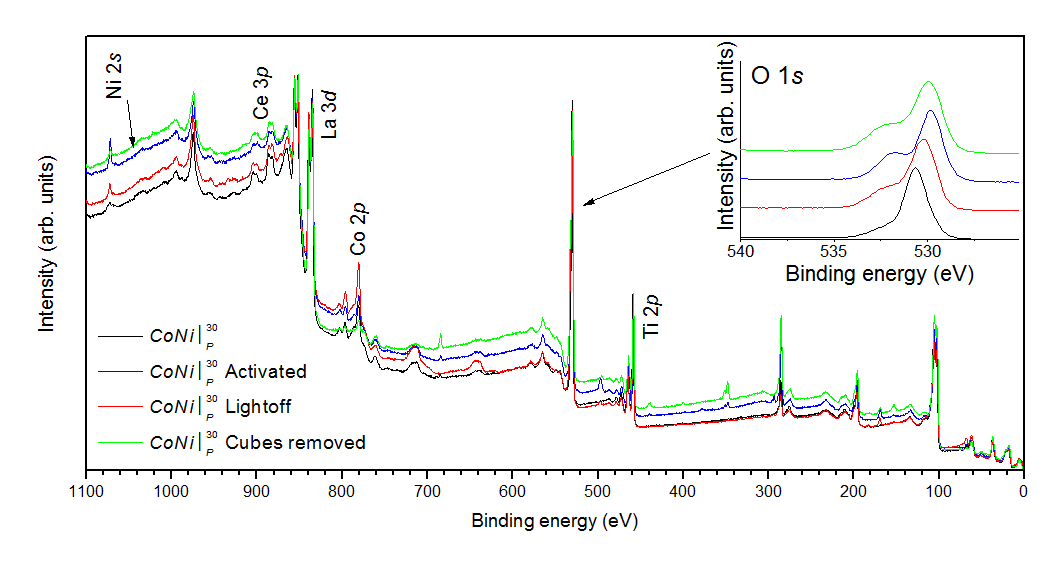 | | |
| **b** | **c** | **d** |
| 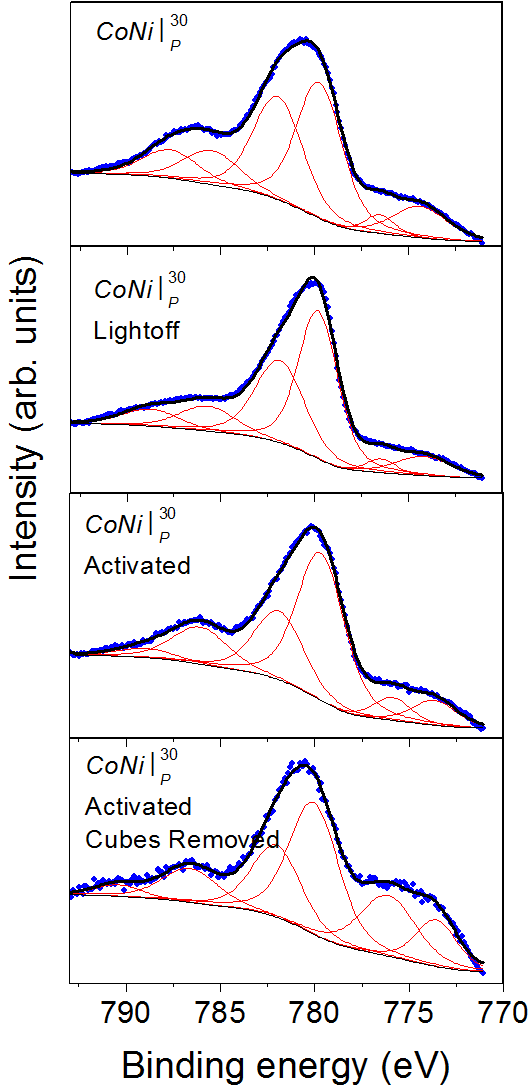 | 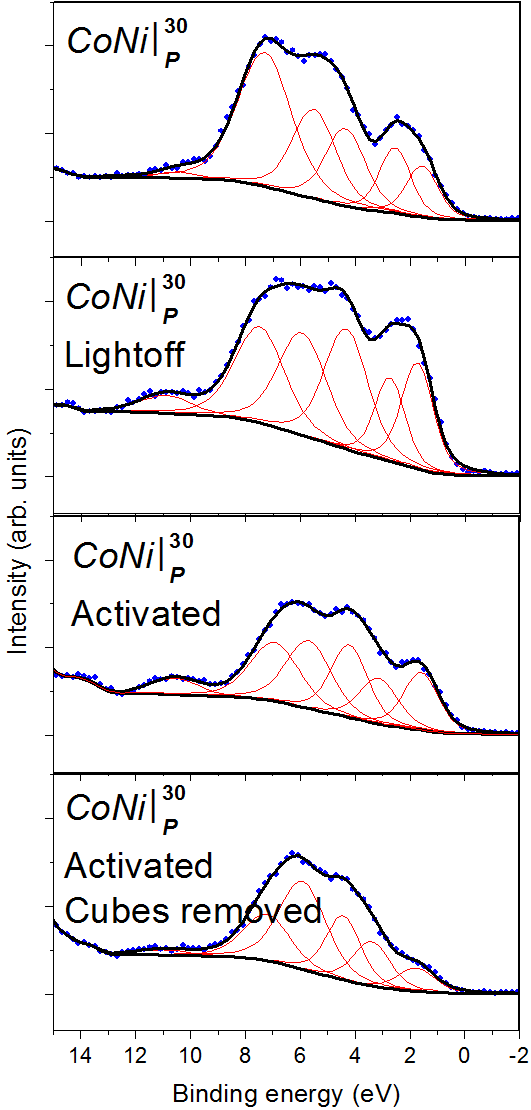 | 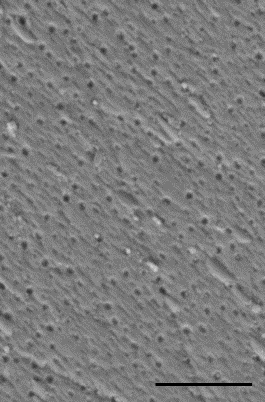 |

Supplementary Figure 5. Additional XPS details for the $\boldsymbol{CoNi}{}_{\boldsymbol{P}}^{\boldsymbol{30}}$sample shown in Fig. 2.

(**a**) Survey also showing detail of the oxygen peak. (**b**) Co 2*p* core-loss spectra. (**c**) valence band spectra. (**d**) the microstructure of the activated samples after cube removal; scale bar, 500 nm. The removal was done with a soft brush, compressed air and gentle cleaning with acetone in an ultrasonic bath. A small number of leftover ‘cubes’ can still be observed, probably accounting for the small amount of leftover Co_3_O_4_ (Fig. 2c). The fits serve mainly as guide to the eye.


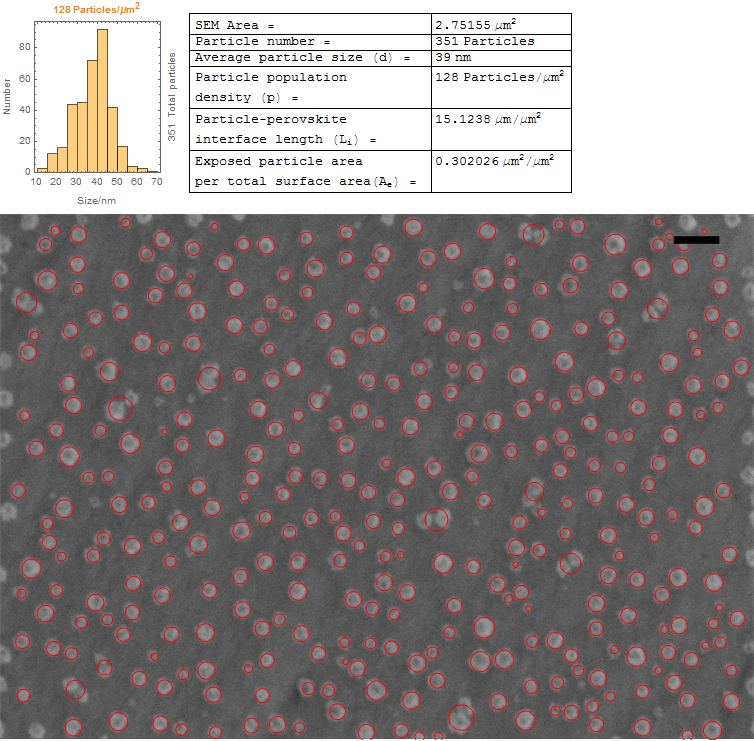


Supplementary Figure 6. The microstructure and particle size analysis of $\boldsymbol{Ni}{}_{\boldsymbol{P}}^{\boldsymbol{30}}$after light-off.

This analysis was performed in the same area as that in Supplementary Fig. 2. The average particle size is 39 nm. Scale bar, 100 nm. The particle size increase is ~(39 nm)/(30 nm) = 1.3, which is reasonably close to the expected expansion from Ni^0^ to NiO, (3.5238 Å)/(4.1752 Å) = 1.18.


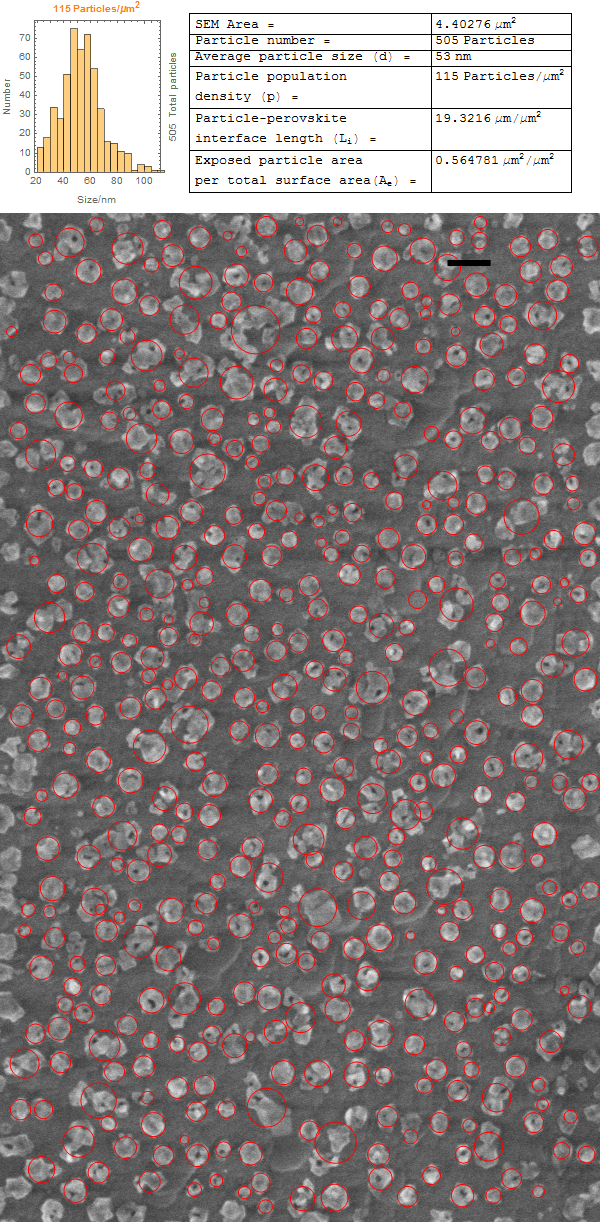


Supplementary Figure 7. The microstructure and particle size analysis of $\boldsymbol{CoNi}{}_{\boldsymbol{P}}^{\boldsymbol{30}}$ after light-off.

This analysis was done in the same area as that in Supplementary Figure3. Scale bar, 100 nm. The average particle size is 53 nm. Particle size increase, (53 nm)/(30 nm) = 1.76.

| **a** | **b** | **c** |
| --- | --- | --- |
| 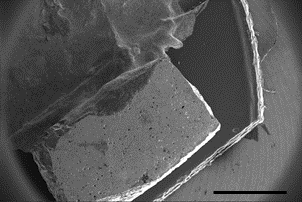 | 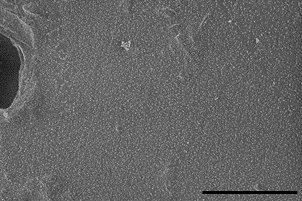 | 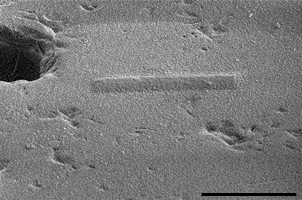 |
| **d** | **e** | **f** |
| 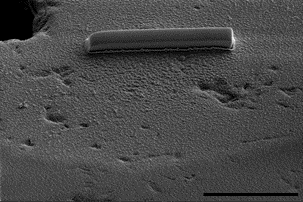 | 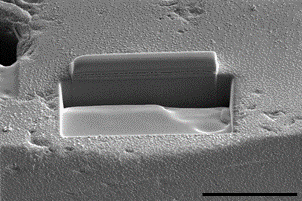 | 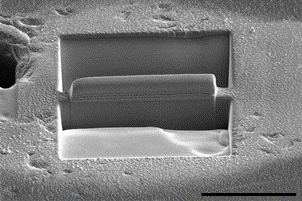 |
| **g** |  | **h** |
| 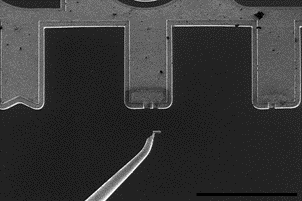 | | 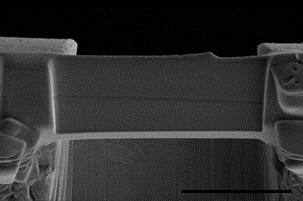 |
|  |  | **i** |
|  |  | 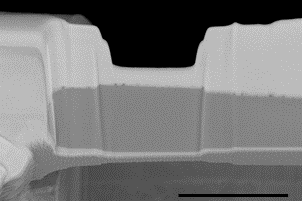 |

Supplementary Figure 8. Preparation of a lamella for TEM by FIB.

(**a**) A small fragment from sample $CoNi{}_{P}^{30}$after activation was grounded with some silver paste; scale bar, 1 mm. (**b**) Detail from the surface of the sample shown in (a); scale bar, 10 µm. (**c**) A thin (< 100 nm), protective layer of carbon was deposited on the area of interest; scale bar, 10 µm. (**d**) A layer of Pt (~1 μm) was then deposited; scale bar, 10 µm. (**e**) and (**f**) trenches were formed on either sides of the area of interest with the help of the ion beam; scale bar, 10 µm. (**g**) the lamella was then cut loose, mounted on a Pt needle and docked into a TEM copper grid; scale bar, 200 µm. (**h**) the lamella as mounted on the TEM grid; scale bar, 5 µm. (**i**) the lamella after thinning and polishing with the focused ion beam; the surface features in the sample become apparent; scale bar, 3 µm.

| **a** | **b** |
| --- | --- |
| 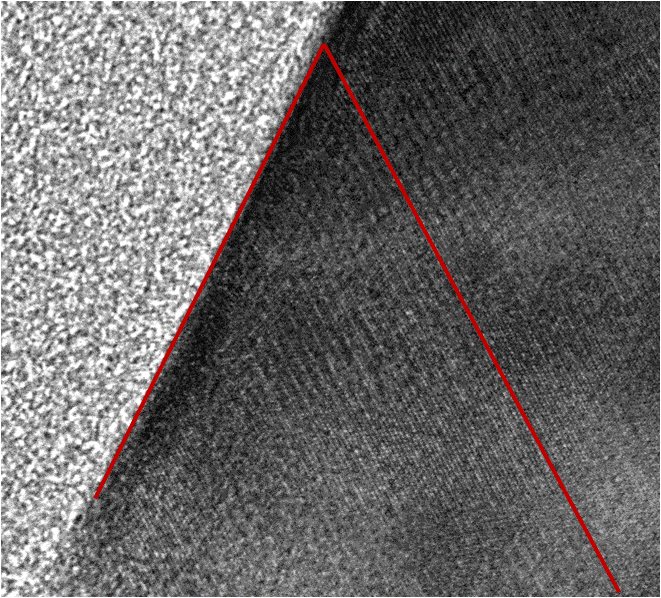 | 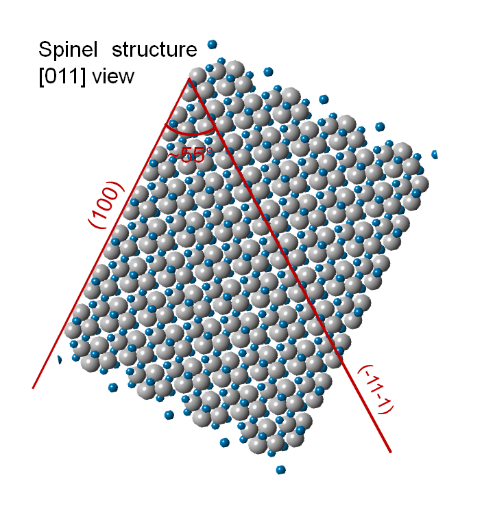 |
| **c** | |
| 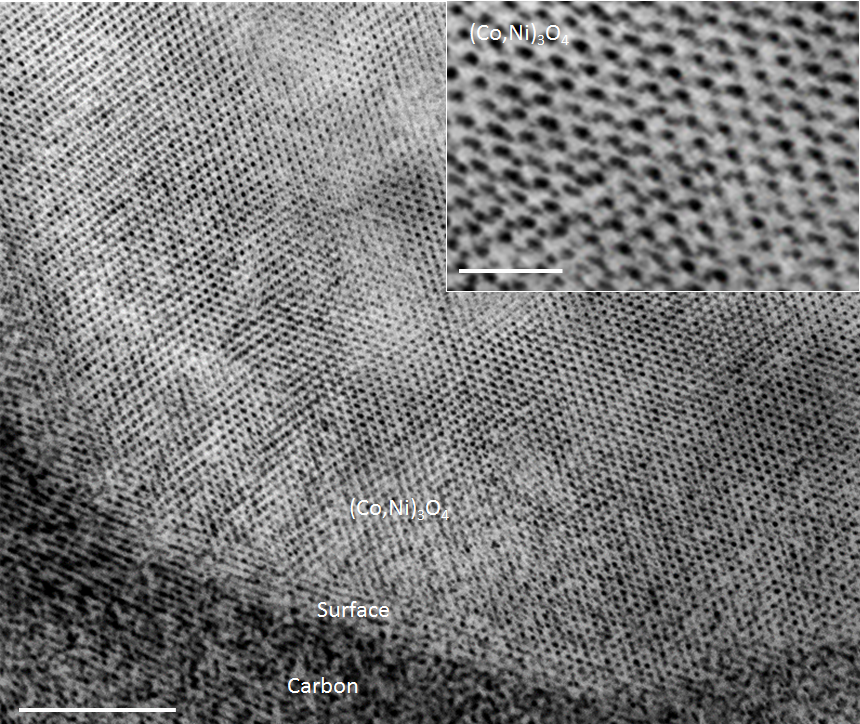 | |

Supplementary Figure 9. STEM and crystal model of the cubes.

(**a**) Detail from Fig. 3c highlighting the angle between the surface of the cube and the observed atomic planes. (**b**) Spinel crystal structure and the orientation corresponding to the A. (**c**) High-resolution TEM from a cube; scale bars, 5 nm and inset, 1 nm.


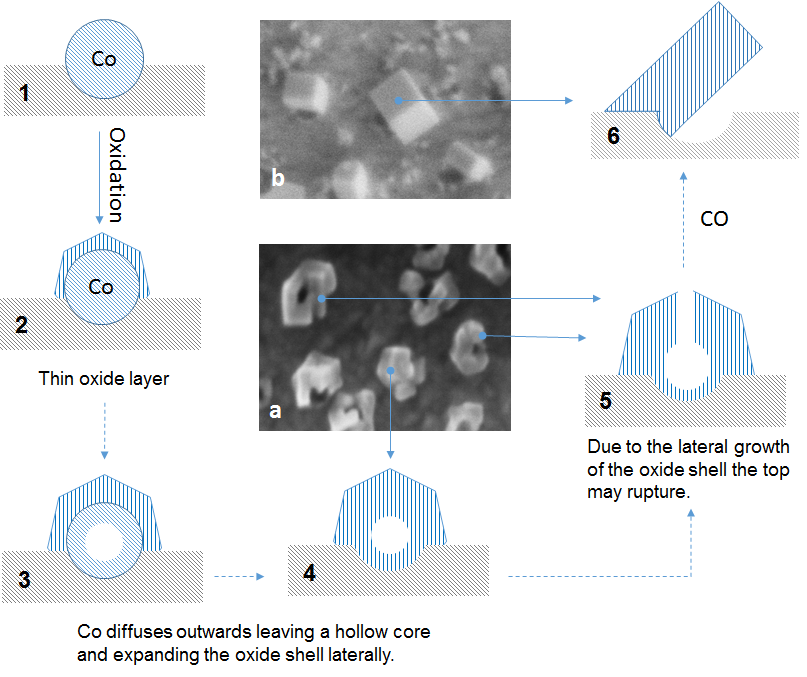


Supplementary Figure 10. Schematic mechanism for the oxidation of exsolved Co-based particles.

(1-6) schematic illustrations. (**a**) Detail from $CoNi{}_{P}^{30}$ after light-off. (**b**) Detail from $CoNi{}_{P}^{30}$after activation during CO kinetics.


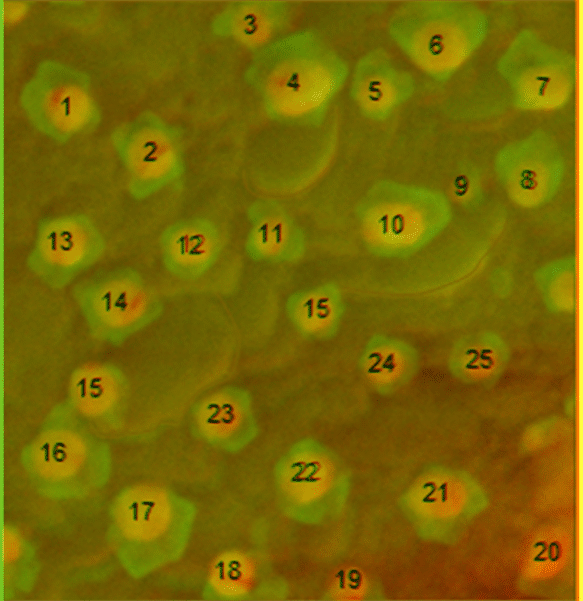


Supplementary Figure 11. False-color image of superimposed micrographs in Fig. 2f and 2g. Yellow indicates the initial metal particles and the green hue the expanded oxide particle.


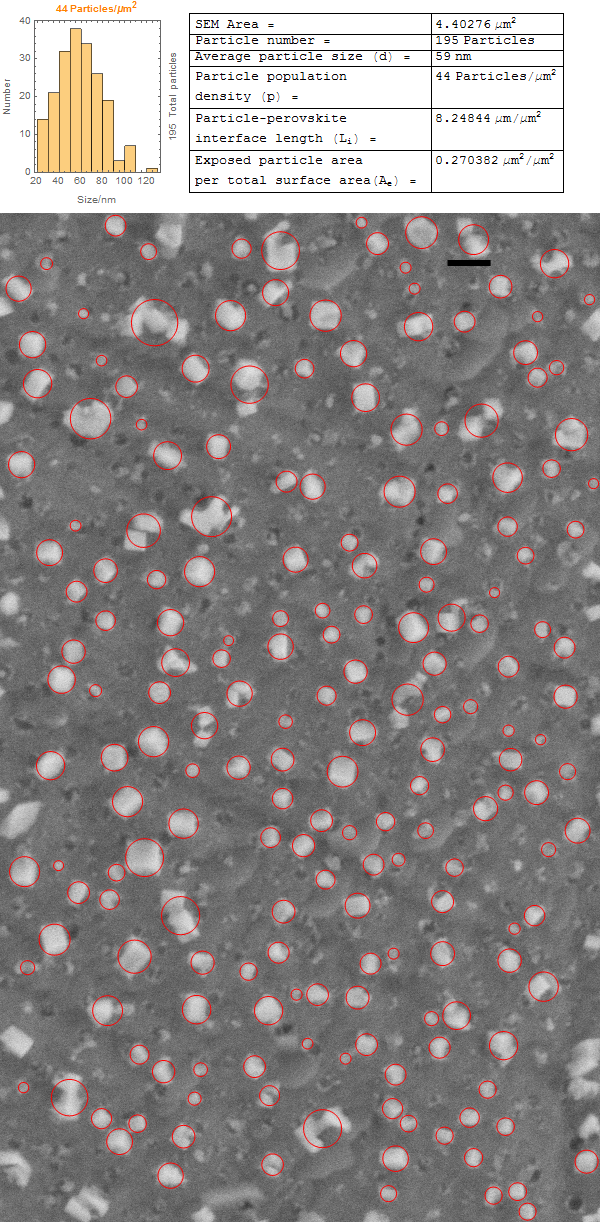


Supplementary Figure 12. The microstructure and particle size analysis of $\boldsymbol{CoNi}{}_{\boldsymbol{P}}^{\boldsymbol{30}}$after activation.

This analysis was done in the same area as that in Supplementary Fig. 3 and 7. The average particle size is 59 nm. Scale bar, 100 nm.

| **a** |
| --- |
| 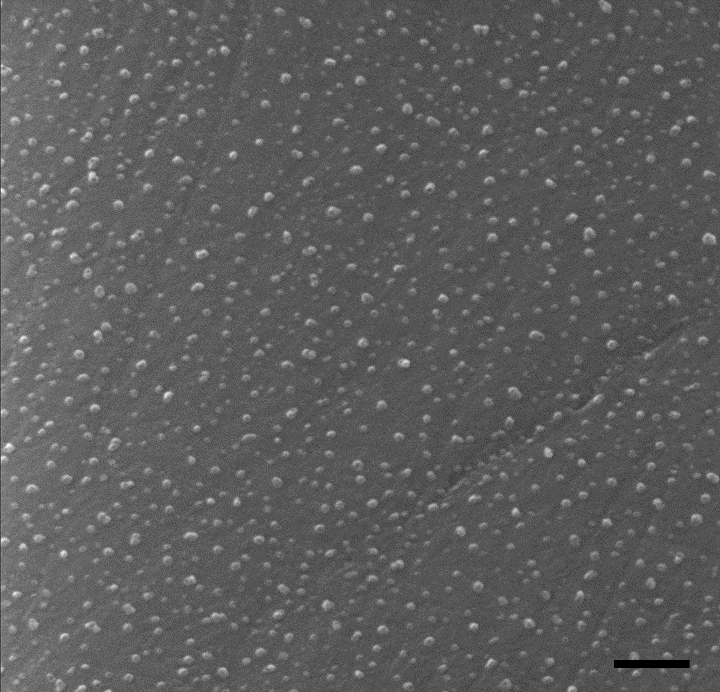 |
| **b** |
| 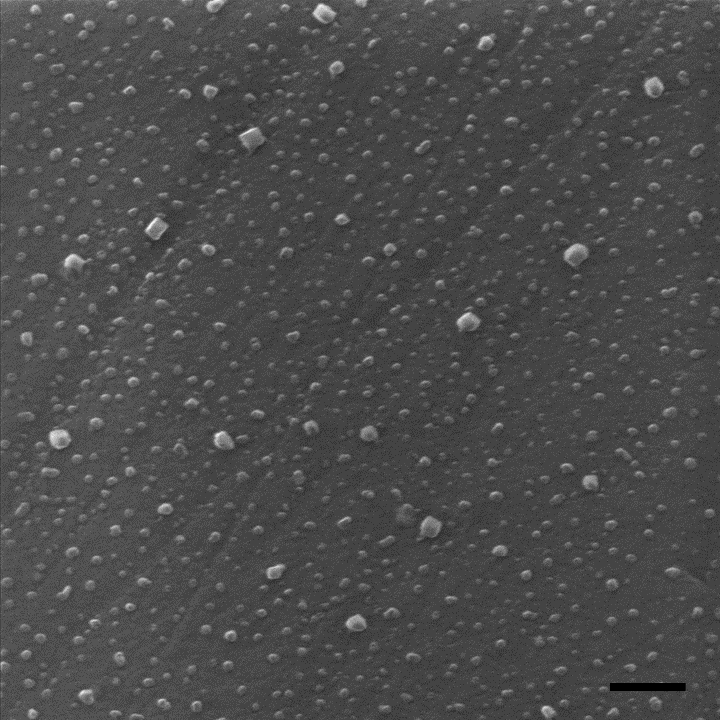 |

Supplementary Figure 13. Microstructure of the $\boldsymbol{CoNi}{}_{\boldsymbol{P}}^{\boldsymbol{10}}$ in Supplementary Figure4 after further treatment.

(**a**) After light-off. (**b**) Sample shown in A after CO kinetics. Scale bars, 100 nm.


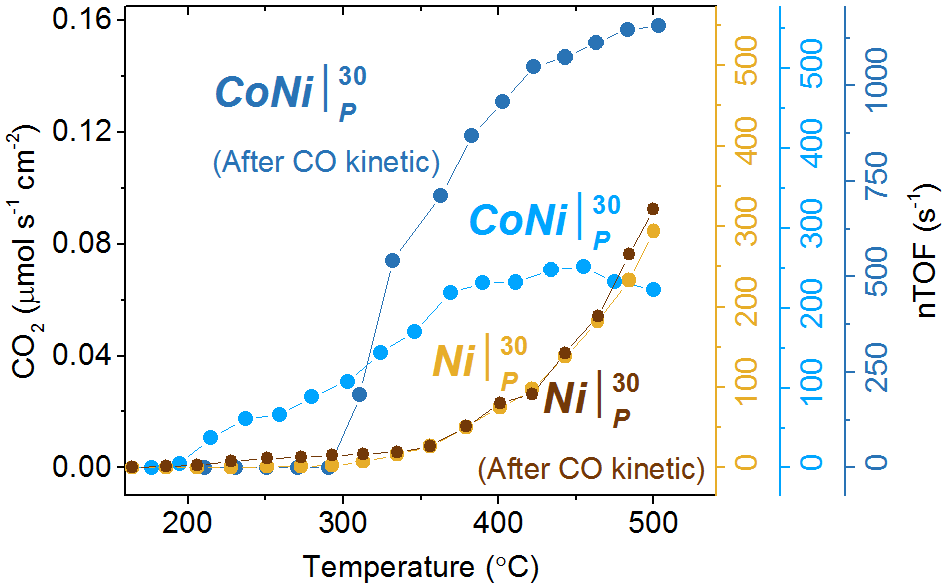


Supplementary Figure 14. Light-off for $\boldsymbol{Ni}{}_{\boldsymbol{P}}^{\boldsymbol{30}}$sample before and after CO kinetics.

This test was carried out on pellet samples. Inlet gas mixture: 1% of O_2_ and 0.6% of CO.


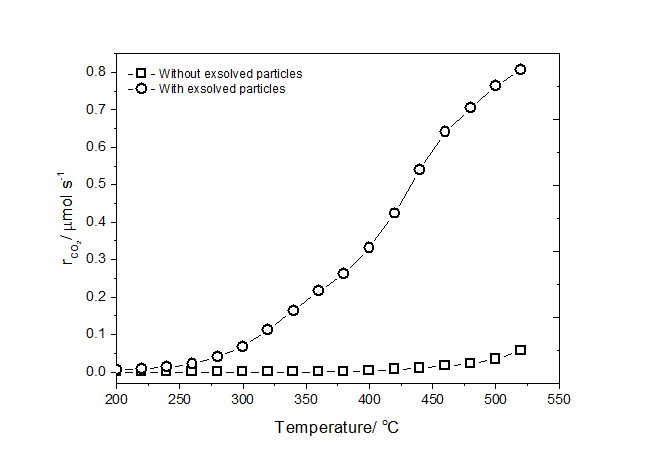


Supplementary Figure 15. Catalytic activity before and after the exsolution of metal particles.

This test was carried out on a La_0.7_Ce_0.1_Co_0.3_Ni_0.1_Ti_0.6_O_3_ powder samples (10 mg of catalyst and 90 mg of Al_2_O_3_). Inlet gas mixture: 1% of O_2_ and 0.6% of CO.


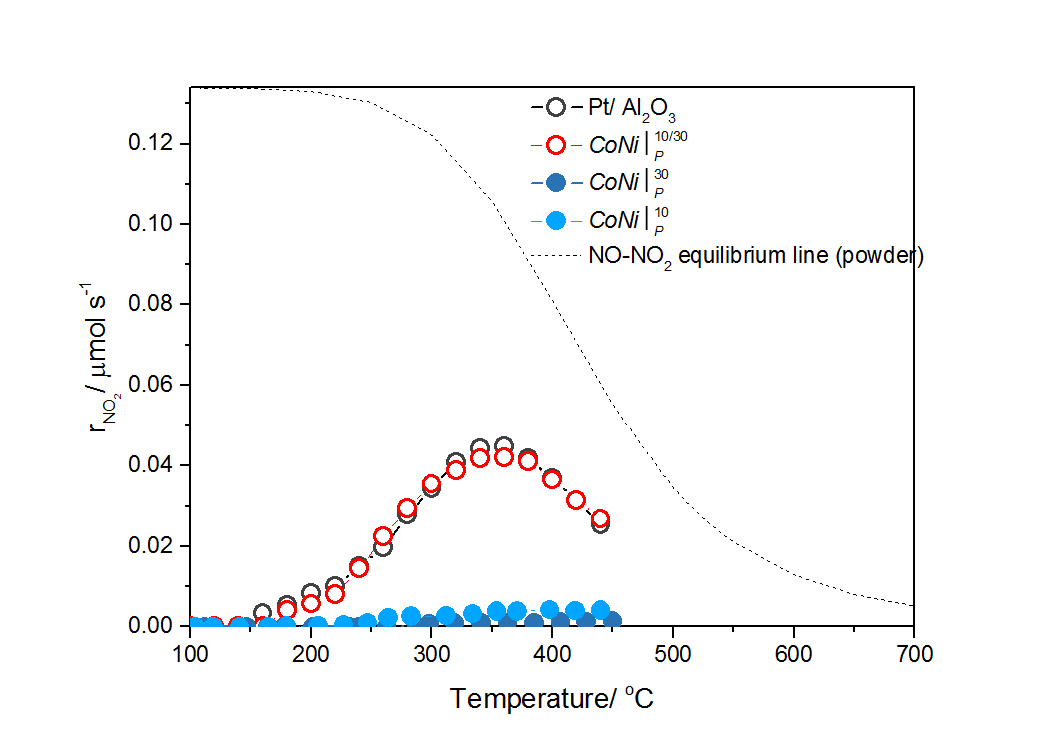


Supplementary Figure 16. NO oxidation and the equilibrium curve.

Inlet gas mixture of 8% of O_2_ and 0.04% of NO.


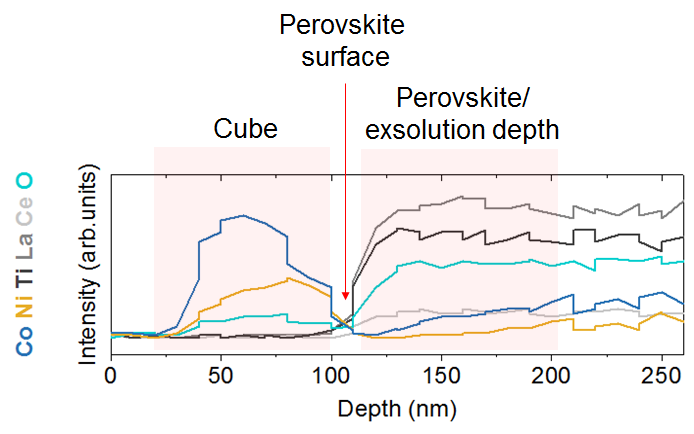


**Supplementary Figure 17. EDX profile across the cube/perovskite interface.** This corresponds to Fig. 3d with additional annotations.

Supplementary Notes

Supplementary Note 1. Structural changes on light-off.

For $Ni{}_{P}^{30}$, the average particle size increase on oxidation is comparable to the expected lattice expansion from Ni^0^ to NiO (see Supplementary Fig. 6). For $CoNi{}_{P}^{30}$ the expansion was considerably larger than expected (see Supplementary Fig. 7) due to hollow core particle formation, as discussed in the main text. Considering that the relative lattice expansion from metal to oxide phase is considerably greater than that experienced by the lattice of the support (whose oxygen content only changes marginally), it follows that due to confinement in the support, the particles are subjected to additional compressive strain upon conducting the light-off.

Supplementary Note 2. Calculation of exsolution amount.

According to Supplementary Figure 17, Co and Ni seem to exsolve from a region that is about 200 nm deep with respect to the surface (note that Co and Ni levels gradually decrease over this depth towards the surface). Corroborating this with the corresponding particle size distribution and particle coverage data for this sample (Supplementary Figure 3) indicates that about 0.06 Co(Ni) atoms per unit cell have exsolved, on average, over this 200 nm depth. In terms of local stoichiometry this equates to a change from La_0.7_Ce_0.1_(CoNi)_0.4_Ti_0.6_O_3_ to La_0.7_Ce_0.1_(CoNi)_0.34_Ti_0.6_O_3_ (=La_0.74_Ce_0.11_(CoNi)_0.36_Ti_0.64_O_3_, if normalised to B-site occupancy 1; note that Ti levels rise slightly as Ni/Co levels fall).

Supplementary Note 3. Calculation of nominal turnover frequency.

Nominal turnover frequency (nTOF) is calculated as the number of molecules reacted per second, per exposed metal atom site at the surface of particles. To calculate nTOFs for our systems we combine kinetic and tracking data and we follow the thought process below.

The number of reacted CO molecules per second is given by the product of the reaction rate ($r_{\text{CO}_{\text{2}}}$) and Avogadro’s number (N_A_), $r_{\text{CO}_{\text{2}}}\cdot N_{A}$.

The exposed particle area in one pellet (i.e. the model catalyst) is obtained by multiplying the area of the pellet surface decorated with particles (*A*) with the exposed particle are per total surface area (*e*), that is, $A_{e}\cdot A$. Where *A_e_* was calculated from SEM micrograph analysis and is listed in the corresponding supplementary figures, such as Supplementary Figure 2.

The corresponding number of active sites will be given by $\left( A_{e}\cdot A \right)/{a^{2}\cdot k}$, where *a* is the unit cell parameter of the crystal lattice of the particles and *k* is the average number of metal sites per unit cell face. The faces were considered to be in a (100) termination, thus, for the NiO rock-salt structure *k* = 1 and for the (Co,Ni)_3_O_4_ spinel structure *k* = 2.

The nTOF is then given by ${N_{A}\cdot r}_{\text{CO}_{\text{2}}}\cdot{a^{2}}/\left( A_{e}\cdot A\cdot k \right)$.

When the various measures are expressed as follows, $r_{\text{CO}_{\text{2}}}$ (mol s^-1^), N_A_ (mol^-1^), *a* (nm), *A_e_* (μm^2^ μm^-2^), *A* (cm^2^), the nTOF (s^‑1^) is given by Supplementary Equation 1:

| $\mathrm{nTOF}={{{10}^{-20}\cdot N}_{A}\cdot r}_{\text{CO}_{\text{2}}}\cdot{a^{2}}/\left( A_{pe}\cdot A_{p}\cdot k \right)$ | (1) |
| --- | --- |

A few representative examples are given in Supplementary Table 1.

**Supplementary Table 1. Calculation of nTOF values.**

|  | $Ni{}_{P}^{30}$ | $CoNi{}_{P}^{30}$ | $CoNi{}_{P}^{30}$ (activated) |
| --- | --- | --- | --- |
| Temperature (°C) | 500 | 500 | 500 |
| CO_2_ production rate  $r_{\text{CO}_{\text{2}}}$ (μmol s^-1^) | 0.154 | 0.118 | 0.294 |
| Pellet area decorated with particles  *A* (cm^2^) | 1.82 | 1.86 | 1.86 |
| Exposed particle area per total surface area  *A_e_* (μm^2^ μm^-2^) | 0.302 | 0.564 | 0.270 |
| *A_e_* given in Supplementary Figure | S6 | S7 | S12 |
| Unit cell parameter of the particles  *a* (nm) | 0.41752 | 0.80835 | 0.81000 |
| Exposed metal sites per unit cell  *k* (atoms per unit cell) | 1 | 2 | 2 |
| Nominal turnover frequency  nTOF (s^-1^) | 294.2 | 221.3 | 1156.7 |

For the Pt/Al_2_O_3_ powder catalyst, the nTOF is given by Supplementary Equation 2:

| $\mathrm{nTOF}={{{10}^{-24}\cdot N}_{A}\cdot r}_{\text{CO}_{\text{2}}}\cdot{a^{2}}/\left( A_{e}\cdot k \right)$ | (2) |
| --- | --- |

At 250 °C, $r_{\text{CO}_{\text{2}}}$= 25 μmol s^-1^ g^-1^ (Fig. 4A), *a* = 0.3912 nm, *A_e_* = 1 m^2^ g^-1^, k = 2, which results in nTOF = 1.1 s^-1^.

Supplementary Methods

**Perovskite pellet preparation**

The perovskite oxides were prepared by a modified solid state synthesis. High purity precursors including La_2_O_3_ (Pi-Kem, >99.99%), CeO_2_ (Alfa Aesar, >99.99%), TiO_2_ (Alfa Aesar, >99.6%), Co_3_O_4_(Aldrich, >99.5%) and Ni(NO_3_)_2_*6H_2_O (Acros, >99%) were used in the appropriate stoichiometric ratios. Certain oxides were dried at different temperatures (TiO_2_ - 300 °C, La_2_O_3_ - 800 °C) and weighed while warm. The reaction mixture was quantitatively transferred to a beaker and mixed with acetone and ~0.05 wt.% Hypermer KD1 dispersant. An ultrasonic Hielscher UP200S probe was used to break down agglomerates and homogenize the mixture into a fine, stable dispersion. The acetone was then evaporated at room temperature under continuous stirring and the content of the beaker was quantitatively transferred to a crucible and calcined at 1000 °C for 12 hours. The calcined powder was then pressed into 20 mm diameter pellets (~1.5 g of powder per pellet) and fired at 1390 °C for 16 hours to form the perovskite phase in dense pellet form (relative density >95%). The as-prepared pellets were further processed for the tracking experiments as explained below, or for the preparation of the powder, they were crushed and ball-milled to produce a powder with a total specific surface area of ~1.5 m^2^ g^‑1^.

**Catalytic tests**

For the catalytic experiment with the pellets a continuous-ﬂow single-chamber reactor with a total gas-phase volume of 30 cm^3^ was used. Upstream to this reactor a fixed-bed reactor with alumina powder heated at 90 ^o^C was used to capture possible carbonyl species. Electronic mass ﬂow controllers (MFCs) were used to control the ﬂow of the feeding gas to the reactor. All experiments were conducted at atmospheric pressure. A K-type thermocouple was used to measure the sample temperature. The thermocouple was placed in proximity to the pellet surface. During the light-off experiment, the maximum temperature difference between the sample temperature and the gas temperature cannot be higher than 15 ^o^C for the non-activated pellet samples and 30 ^o^C for the activated pellet samples (assuming a value of 10 W m^-2^ K^-1^ for the heat transfer coefficient between the sample and the gas phase, a conservative value appropriate for natural convection or low gas velocities and a heat of reaction approximately 280 kJ mol^-1^). The gases used were 20% CO/He, 20% O_2_/He, and CP grade He (N5) provided by BOC Ltd. Flow rates of 1 × 10^−4^ mol s^−1^ (150 cm^3^ min^−1^) were used (volumetric flow rates are given at normal temperature and pressure (NTP)). The ﬂow rates were also measured at the outlet using a Varian digital ﬂow meter (1000 series). Helium was used as a balance gas throughout the experiments.

An XTREAM-CO_2_ analyzer provided by Rosemount was used to analyze the carbon dioxide (CO_2_) mole fraction in the product stream. The minimum detectable CO_2_ mole fraction for the XTREAM-CO_2_ analyzer was 1 ppm, which corresponds to a minimum measurable rate of CO_2_ production of 1 × 10^−10^ mol s^−1^ with typical ﬂow rate at 1 × 10^−4^ mol s^−1^ (150 cm^3^ min^−1^). Reaction rates ($r_{\text{CO}_{\text{2}}}$) in terms of CO_2_ production are calculated as shown in Supplementary Equation 3:

$r_{\text{CO}_{\text{2}}}\left( \text{mol(C}\text{O}_{\text{2}}\text{)}\text{ }\text{s}^{-1}\text{m}^{-2} \right)=y_{\text{CO}_{\text{2}}}\cdot\dot{n}\cdot A^{-1}$ (3)

where $y_{\text{CO}_{\text{2}}}$ is the measured CO_2_ mole fraction at the gas outlet, ṅ is the molar ﬂow, and A is the top side pellet area. In order to be able to measure the rate of CO_2_ production (and the nTOFs, see Supplementary Note 1) during the light-off experiment under ‘gradientless’ conditions the reactor was operated under conditions of differential conversion (20% conversion of CO or less for the non-activated pellet samples and 40% conversion of CO or less for the activated pellet samples).

To study the eﬀect of temperature, the pellets were heated in an inlet gas mixture of 1% of O_2_ and 0.6% of CO from 100 °C up to 520 °C. The temperature was held during heating after each step of 20 °C, the holding time being varied depending on the time the reaction rate needed to become steady: i.e., the rate of CO_2_ production did not vary by more than ±5% over 60 min. The heating rate between the isothermal periods was 2 °C min^−1^. To study the influence of CO the O_2_ inlet mole fraction was held constant at 0.64% and the CO inlet mole fraction was allowed to vary between 0.5 and 18.9%.

For the catalytic experiment with the powders a fix packed-bed reactor was used. Upstream to this reactor a fixed-bed reactor with alumina powder heated at 90^o^C was used to capture possible carbonyl species. Volumetric dilution within catalyst bed is made by mixing the catalyst powder with Al_2_O_3_ powder (10 wt.% of each catalyst is diluted with Al_2_O_3_ to get a total weight of 100 mg. It was found that this amount of catalyst sample mixed with Al_2_O_3_ resulted in a reasonable pressure drop within the bed). Electronic mass ﬂow controllers (MFCs) were used to control the ﬂow of the feeding gas to the reactor. All experiments were conducted at atmospheric pressure. A K-type thermocouple was used to measure the sample temperature. The thermocouple was placed outside the bed in proximity to the sample. The gases used were 20% CO/He, 20% O_2_/He, 1% NO/He, and CP grade He (N5) provided by BOC Ltd. Flow rates of 3 × 10^−4^ mol s^−1^ (450 cm^3^ min^−1^, gas hourly space velocity, i.e the volumetric flow rate of the reacting gases divided by the fixed bed volume (GHSV) = 26000 h^-1^) were used (volumetric flow rates are given at normal temperature and pressure (NTP)). The ﬂow rates were also measured at the outlet using a Varian digital ﬂow meter (1000 series). Helium was used as a balance gas throughout the experiments.

For the catalytic experiments with CO an XTREAM-CO_2_ analyzer provided by Rosemount was used to analyze the CO_2_ concentration in the product stream. The minimum detectable CO_2_ mole fraction for the XTREAM-CO_2_ analyzer was 1 ppm, which corresponds to a minimum measurable rate of CO_2_ production of 3 × 10^−10^ mol s^−1^ with typical ﬂow rate at 3 × 10^−4^ mol s^−1^ (450 cm^3^ min^−1^). Reaction rates ($r_{\text{CO}_{\text{2}}}$) in terms of CO_2_ production are calculated as shown in Supplementary Equation 4:

$r_{\text{CO}_{\text{2}}}\left( \text{mol(C}\text{O}_{\text{2}}\text{)}\text{ }\text{s}^{-1} \right)=y_{\text{CO}_{\text{2}}}\cdot\dot{n}$ (4)

where $y_{\text{CO}_{\text{2}}}$ is the measured CO_2_ mole fraction at the gas outlet, ṅ is the molar ﬂow. To study the effect of temperature and the influence of the CO similar steps to the experiments with CO with the pellets were followed. During the light-off experiment the goal was to acquire a weight-to-weight comparison of the activity of the catalysts. The rates extracted are average rates since the composition of the reaction mixture varies along the bed. The nominal temperature of the bed and the composition of the inlet feed gas was known. The inlet feed gas composition did not change during the experiment. In order to be able to measure the rate of CO_2_ production during the long-term experiment (over 650 hours) under gradientless conditions the reactor was operated under conditions of differential conversion (20% conversion of CO or less for both catalysts used).

For the catalytic experiments with NO an XTREAM-NO_x_ X2GP analyzer provided by Rosemount was used to analyze the NO and NO_2_ in the product stream. The minimum detectable NO and NO_2_ mole fraction for the XTREAM-NO_x_ analyzer was 1 ppm, which corresponds to a minimum measurable rate of CO_2_ production of 3 × 10^−10^ mol s^−1^ with typical ﬂow rate at 3 × 10^−4^ mol s^−1^ (450 cm^3^ min^−1^, GHSV = 26000 h^-1^). Reaction rates ($r_{\text{NO}_{\text{2}}}$) in terms of NO_2_ production are calculated as shown in Supplementary Equation 5:

$r_{\text{NO}_{\text{2}}}\left( \text{mol(N}\text{O}_{\text{2}}\text{)}\text{ }\text{s}^{-1} \right)=y_{\text{NO}_{\text{2}}}\cdot\dot{n}$ (5)

where $y_{\mathrm{NO}_{2}}$ $Y_{\text{CO}_{\text{2}}}$ is the measured NO_2_ mole fraction at the gas outlet, ṅ is the molar ﬂow.

To study the eﬀect of temperature, the powders were heated in an inlet gas mixture of 8% of O_2_ and 0.04% of NO from 100 °C up to 440 °C. The temperature was held during heating after each step of 20 °C, the holding time being varied depending on the time the reaction rate needed to become steady: i.e., the rate of NO_2_ production did not vary by more than ±5% over 60 min. The heating rate between the isothermal periods was 2 °C min^−1^. In order to be able to measure the rate of NO_2_ production during the light-off experiment under gradientless conditions the reactor was operated under conditions of differential conversion (35% conversion of NO or less for both catalysts used).
